# Passive Muscle-Tendon Unit Gearing Is Joint Dependent in Human Medial Gastrocnemius

**DOI:** 10.3389/fphys.2016.00095

**Published:** 2016-03-15

**Authors:** Emma F. Hodson-Tole, James M. Wakeling, Taylor J. M. Dick

**Affiliations:** ^1^School of Healthcare Science, Manchester Metropolitan UniversityManchester, UK; ^2^Department of Biomedical Physiology and Kinesiology, Simon Fraser UniversityBurnaby, BC, Canada

**Keywords:** ultrasound imaging, skeletal muscle, force-length relationship, fascicles, bi-articular, biomechanics, anatomy

## Abstract

Skeletal muscles change length and develop force both passively and actively. Gearing allows muscle fiber length changes to be uncoupled from those of the whole muscle-tendon unit. During active contractions this process allows muscles to operate at mechanically favorable conditions for power or economical force production. Here we ask whether gearing is constant in passive muscle; determining the relationship between fascicle and muscle-tendon unit length change in the bi-articular medial gastrocnemius and investigating the influence of whether motion occurs at the knee or ankle joint. Specifically, the same muscle-tendon unit length changes were elicited by rotating either the ankle or knee joint whilst simultaneously measuring fascicle lengths in proximal and distal muscle regions using B-mode ultrasound. In both the proximal and distal muscle region, passive gearing values differed depending on whether ankle or knee motion occurred. Fascicle length changes were greater with ankle motion, likely reflecting anatomical differences in proximal and distal passive tendinous tissues, as well as shape changes of the adjacent mono-articular soleus. This suggests that there is joint-dependent dissociation between the mechanical behavior of muscle fibers and the muscle-tendon unit during passive joint motions that may be important to consider when developing accurate models of bi-articular muscles.

## Introduction

Skeletal muscles are an organ whose primary function is to produce force and hence movement. They are a structurally organized, composite material containing multiple contractile elements, internal and external connective tissues as well as nerve and vascular tissues. They often span two or more joints and many reports have evidenced different geometric arrangements of fibers both between and within individual muscles (for a review see: English et al., 1993); differences in connective tissue properties at sites of origin and insertion (Dalmau-Pastor et al., 2014) and complex myofascial connections between groups of muscles (Ahn et al., 2003; Higham and Biewener, 2011).

The forces developed within a muscle-tendon unit (MTU) during contraction are in part dependent upon the activation state, length, velocity, and orientation of the contracting fibers (Hill, 1938; Katz, 1939). The force-velocity relationship of muscle fibers shows that slower shortening velocities result in higher force and mechanical power production is reduced at higher velocities (Hill, 1938), suggesting a mechanical/energetic advantage may be gained if mechanisms to prevent fibers operating at very high shortening velocities during completion of a motor task are present.

**Figure 1:**
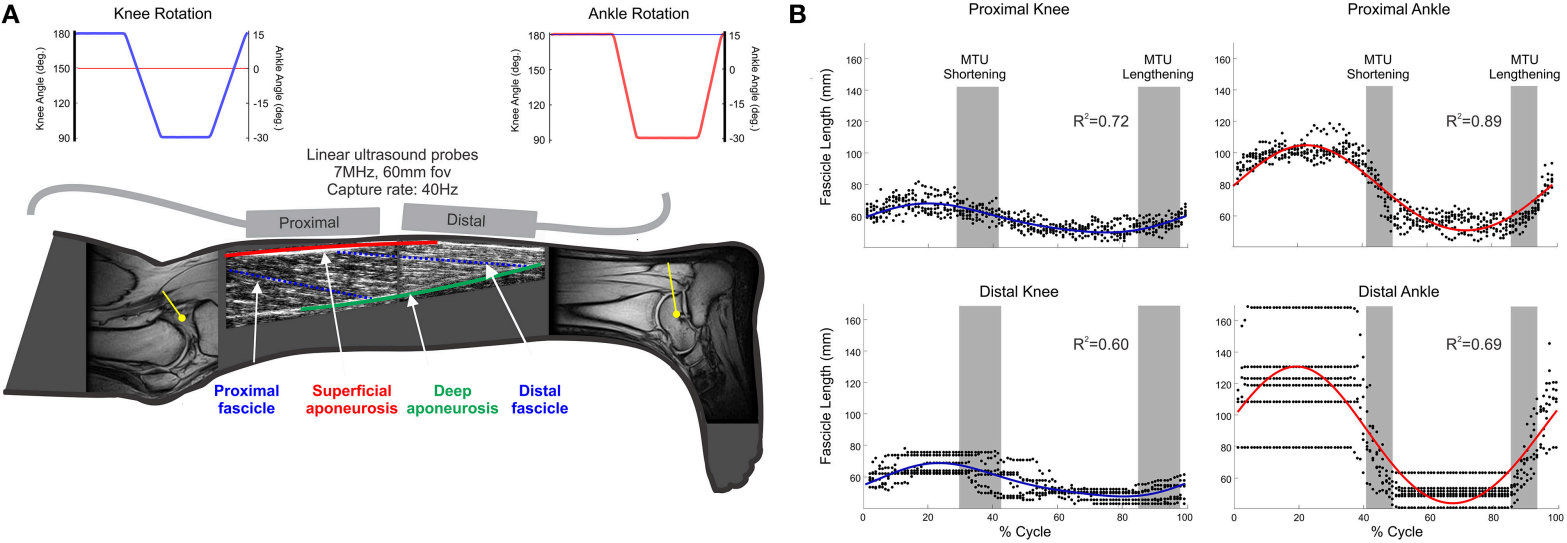
**(A)** Schematic of collected data. (i) MR images of knee and ankle joints, which were used to provide participant-specific moment arm (yellow lines) used in computer simulation to predict MTU lengths; (ii) ultrasound images from proximal and distal MG regions, providing fascicle (blue, broken lines) length (*L*_f_), and pennation; (iii) joint angles during knee (blue) and ankle (red) rotation conditions (Top of panel); **(B)** Representative fascicle length data from one participant. Individual points (black dots) indicate fascicle length at each time point across all recorded movement cycles; solid lines (blue/red) indicate the two-harmonic Fourier series fit to these data with associated *R*^2^ values displayed.

In muscles with pennate fascicle architecture, the fascicles (i.e., fiber bundles) are arranged obliquely to the mid-line of the muscle (Figure 1A). As the muscle belly shortens, the fascicles both shorten and rotate to greater pennation angles. Such fiber rotation means that the fiber velocity (*V*_f_) can be uncoupled from that of the muscle tendon unit (*V*_MTU_): a process termed MTU gearing (Wakeling et al., 2011).

In active contractions variable MTU gearing (*V*_MTU_/*V*_f_) is a well-documented phenomenon, and suggests the presence of underlying mechanisms that enable fibers to shorten at optimal velocities for power production across a range of MTU velocities (Azizi et al., 2008; Wakeling et al., 2011). Variations in gearing are likely to be driven by the pattern of muscle activation which influences the forces developed within the muscle and underpins an ability for an individual muscle to complete tasks with different mechanical demands (Higham et al., 2008).

Muscles, however, also change length and generate forces passively. During many cyclical motor tasks (e.g., stepping, stair negotiation, cycling), there are alternating phases of muscle activation and phases of quiescence. During quiescence, MTU and fiber length changes still occur as joint angles are modified by the action of antagonistic and/or synergistic muscles or external loads. Without active forces being developed within the muscle, passive MTU gearing could be influenced by the kinematics of the movement with implications for the force generating ability of the muscle during the initial period of activation (i.e., influence fiber operating length at the onset of activation).

In humans the medial gastrocnemius muscle (MG) is a bi-articular plantar flexor of the foot which spans both the knee and ankle joints. It has a simple uni-pennate fascicle architecture (Wolf and Kim, 1997), with fascicles tending to be shorter and more pennate in the more proximal region of the muscle (Narici et al., 1996). It originates from a short tendon attached to the medial epicondyle of the femur and inserts via the long Achilles tendon onto the calcaneus. Dynamic changes in MG fascicle geometric properties can also be studied relatively easily using B-mode ultrasound imaging (Namburete et al., 2011; Rana and Wakeling, 2011; Darby et al., 2013). Previous work, exploring the relationship between fascicle and MTU length changes in MG, has shown that during passive ankle joint rotation only a small proportion (~27–30%) of the total MTU length change is reflected in the muscle fascicles (Herbert et al., 2002, 2011, 2014), which would lead to MTU gearing values greater than one. MG is however a bi-articular muscle (spanning both knee and ankle joint) and, as such, does undergo passive length changes as a result of movement of either the joints spanned (Tian et al., 2012).

In addition to the influence of joint kinematics, the tendons of origin and insertion of MG have very different characteristics. Specifically, the MG tendon of origin is much shorter and thicker than the long Achilles tendon, which is also shared with the synergistic soleus and lateral gastrocnemius muscles (Dalmau-Pastor et al., 2014). MTU gearing may therefore differ between passive ankle and knee joint rotation due to the influence of these tendinous structures. Further to this, given the material properties of MG and potential for epimuscular force transmission (Huijing, 2009), fascicle length changes in proximal and distal regions are likely to be affected differently by ankle or knee joint rotation. This could lead to regional variation in MTU gearing. To our knowledge no-one has previously reported passive MTU gearing in a bi-articular muscle resulting from independent rotation of each of the joints spanned. The purpose of this study was therefore to: (i) test whether fascicle and MTU velocity were uncoupled during passive movements of human MG; (ii) determine if MTU gearing varied in MG, depending on the joint at which motion occurred.

## Methods

### Overview of experimental approach

To test whether MTU gearing in MG was joint dependent, a combination of simulation and experimental work was completed with methods selected to provide: (i) estimation of MTU lengths that occurred within a range of *both* knee and ankle joint angles; and (ii) measures of fascicle geometric properties (e.g., fascicle length and pennation angle) that occurred for equivalent MTU lengths within the defined joint angle ranges of motion. The objective was to determine proximal and distal fascicle geometric property changes for the same MTU length change in both ankle and knee joint rotation conditions and hence calculate MTU gearing in each muscle region.

### Participants

Eight healthy volunteers (six male, mean ± S.D. Age: 27.5 ± 1.9 years; height: 175.48 ± 7.16 cm; mass: 70.44 ± 7.44 kg) gave written informed consent to take part in the study, which was approved by Manchester Metropolitan University Local Ethics Committee and complied with principles laid down by the *Declaration of Helsinki*.

### Muscle-tendon unit length estimation

For bi-articular muscles, the MTU length change is determined by the angle of the two joints spanned and their respective moment arms. Participant specific knee and ankle moment arm and shank length measures were therefore used to scale a kinematics model in computer simulation software (Delp et al., 2007). The model was used to estimate the relationship between MTU length and joint angle and enable identification of the ranges of knee and ankle joint angles that resulted in the same range of MTU lengths, which were subsequently used in the experimental protocol. Specifically, MTU length as a function of knee joint angle was calculated with ankle joint considered to be fixed at 0° (foot segment perpendicular to the tibia), while MTU length as a function of ankle joint angle was calculated with the knee joint considered fixed at 180° (full extension).

To determine proximal and distal MG moment arms, participants lay supine on the bed of a MRI scanner (0.25 Tesla, Esaote, G-Scan, Genova, Italy), with their right leg inserted into a coil, positioned to image either the ankle or knee joint. Axial images (T1-weighted spin echo protocol: repetition time: 580 ms; echo time: 16 ms; number of excitations: 1·0, field of view: 150 × 200 mm; slice thickness: 5 mm; gap between slices: 5.5 mm) were collected (Figure 1A). Once images had been collected from one joint, the participant was repositioned and the protocol repeated for the second joint, with the order of joint positions randomized between participants.

Three MR images of the ankle joint (15° plantar flexion; 15° dorsiflexion; neutral) were used to estimate the joint center of rotation using a geometric approach. The moment arm was calculated as the perpendicular distance from this joint center to the Achilles tendon, as described by Reuleaux (1875) and reported in Maganaris et al. (1998a). Knee moment arms were calculated, from a single image, as the perpendicular distance from the intersection of the anterior and posterior cruciate ligaments to the MG proximal tendon (O'connor et al., 1989; Figure 1A).

### Measurement of muscle characteristics

Participants lay prone on an isokinetic dynamometer (HUMAC®/NORM™ 770, Computer Sports Medicine Inc., Stoughton, MA, USA), with their right leg aligned to enable angular changes of either the ankle or knee joint (Figure 1A). Two linear ultrasound probes (LogicScan 128 and Echo Blaster 128, Telemed, Vilnius, Lithuania) were aligned to the fascicle plane in the proximal and distal regions of MG and held in place using elasticized bandage (Coban™, 3M™ Bracknell, UK).

Passive joint rotations, cycling between the pre-defined range of joint angles (ankle: −15° dorsiflexion to +30° plantar flexion, with the knee fixed at 180°; knee: 180° full extension to 90° flexion, with the ankle fixed at 0°, both at 60° s^−1^), were completed following a short warm up period. Three trials were completed before, without moving the participant, the dynamometer was realigned to enable testing of the second joint. During ankle joint rotation the knee was fully extended along the length of the bed of the dynamometer. During knee joint rotation the ankle was fixed at 0° using a customized boot-style brace device (Figure 1A). The order in which the joints were investigated was randomized between participants.

Ultrasound images from each muscle region were collected (40 f.p.s., one PC laptop dedicated to each ultrasound device), while joint angle measures from the dynamometer were collected (2000 Hz) through a 16-bit data acquisition card (USB 6210, National Instruments Corp., Austin, TX, USA) using custom-written software (LabVIEW 2009, National Instruments Corp.). All data sources were synchronized by a common trigger signal.

### Calculation of muscle geometric properties

Ultrasound images from the proximal muscle region were analyzed using an automated Bayesian multiple hypothesis approach that provided the average shape of all MG fascicles visible in each image (Darby et al., 2013). Variation in fascicle orientations in the distal muscle portion meant this approach was not appropriate for use on these images (Figure 1A), so they were manually digitized. Pennation angle (β) in the proximal and distal regions was calculated as the mean of the angles made by the fascicle with the superficial and deep aponeuroses (Figure 1A).

Estimated geometric properties were split into individual movement cycles (e.g., ankle joint rotation from −15° through to −15°) and all cycles of movement from each condition grouped. To provide smoothly evolving data values (and simultaneously acting to filter these data), two-harmonic Fourier series were fit to each geometric property (fascicle length and pennation angle; Figure 1B).

For each participant, based on the combination of their moment arm data and musculoskeletal simulation, a 20 mm range of MTU lengths that occurred for both ankle and knee rotations was identified. Using the identified Fourier series coefficients, muscle geometric properties were calculated for these equivalent regions (i.e., across range of joint angles over which identified 20 mm range of MTU lengths occurred). Values were interpolated to 20 equally spaced values per muscle lengthening or shortening epoch and displayed as a function of MTU length (*L*_MTU_). Predicted fascicle lengths were converted to strains (ε) using a reference length, determined in each muscle region, as the mean fascicle length across all trials. MTU gearing is given as the ratio of MTU velocity to fascicle velocity (*V*_MTU_/*V*_f_). As fascicle and *L*_MTU_ changes were determined across the same time period, this MTU gearing was equivalent to the ratio of their change in lengths (Δ*L*_MTU_/Δ*L*_f_) for each epoch.

### Statistical analyses

In each participant a least squares linear line of best fit was determined for the relationship between MTU length and each geometric property (*L*_f_, β) for the lengthening and shortening phase of the movement cycle. Paired *T*-tests were used to identify significant differences (α = 0.05) in the slope of the best fit line between knee and ankle joint rotations within each phase. Mann Whitney U-tests were performed for nonparametric data, which were identified using Shapiro-Wilk tests.

## Results

### Moment Arm and MTU lengths

The recorded moment arm data, and other anthropometric measures, for each participant are presented in Table 1. Greater moment arm values were recorded at the ankle (49.16 ± 8.77 mm) than at the knee (18.65 ± 1.40 mm). These values resulted in a mean ankle:knee moment arm ratio of: 2.62 ± 0.35. The 20 mm range of *L*_MTU_ over which muscle geometric properties and MTU gearing was calculated started from between 401.34 and 477.03 mm across the group (Table 1).

**Table 1:** **Participant anthropometric data, moment arm (from MRI data), and the shortest *L*_MTU_ from which the 20 mm range were selected for gearing calculations**.

	**Sex**	**Height (cm)**	**Weight (kg)**	**Shank length (cm)**	**Ankle MA (cm)**	**Knee MA (cm)**	**Min MTU length (cm)**
1	F	174.0	62.0	45.7	4.39	1.84	46.38
2	M	179.0	72.5	42.8	5.06	2.04	42.91
3	M	177.5	72.0	45.3	6.07	2.02	45.43
4	F	165.0	63.0	41.0	3.50	1.66	41.54
5	M	180.3	67.0	43.0	5.47	1.78	42.91
6	M	185.0	86.0	47.0	4.10	1.73	47.70
7	M	165.0	70.0	39.8	5.79	1.98	40.13
8	M	178.0	71.0	44.0	4.96	1.86	43.75

*MA, Moment Arm; M, Male; F, Female*.

### Muscle geometric properties

**Figure 2:**
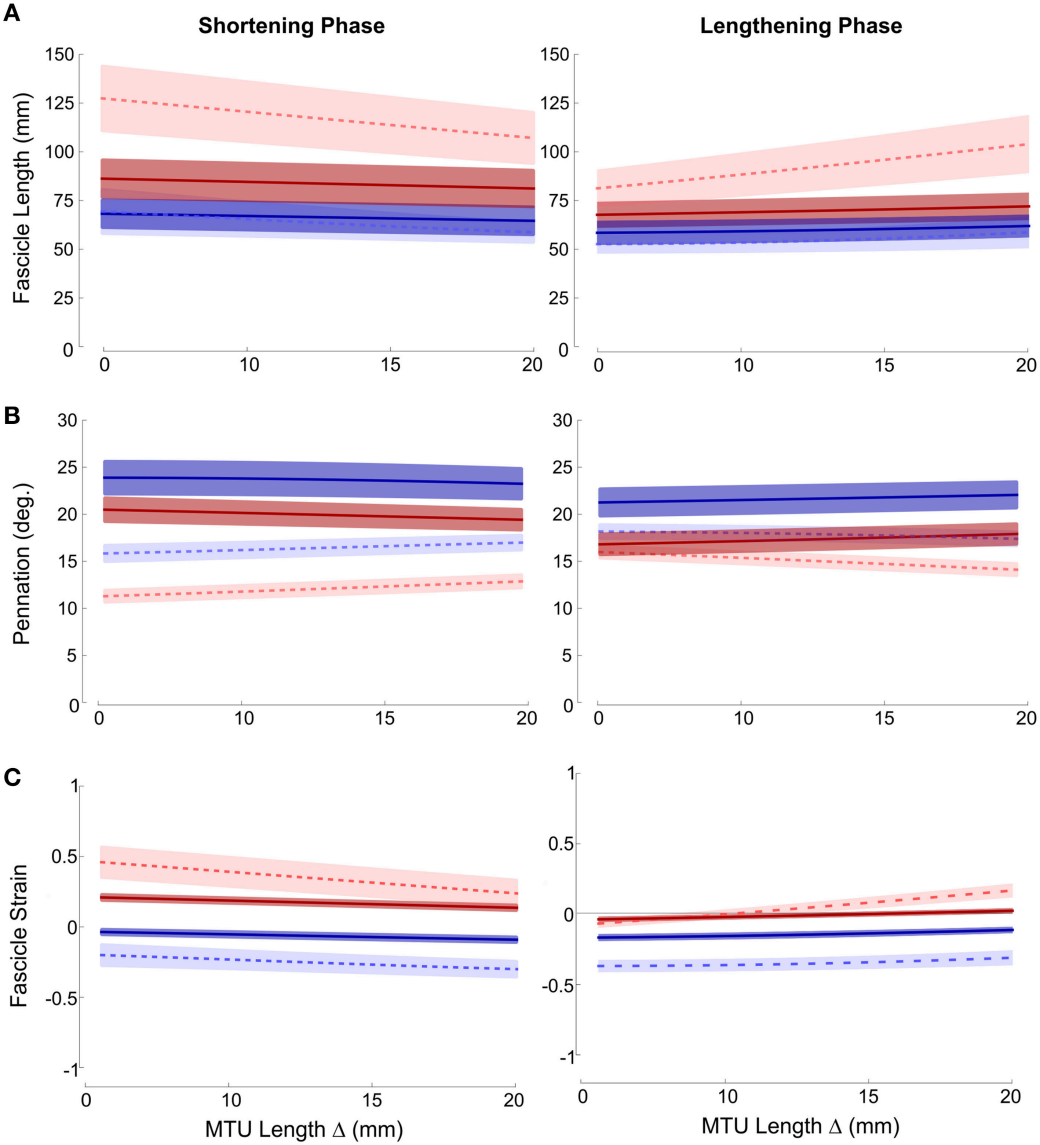
**Mean ± SEM (A) fascicle length; (B) pennation; (C) fascicle strain plotted against change in MTU length**. Proximal (darker, solid lines); distal (lighter, dashed lines); red (ankle movement); blue (knee movement).

Fascicle lengths and pennations as a function of MTU length change are shown in Figures 2A,B, respectively. Large variation in fascicle lengths across the grouped data is evident, reflecting individual variation in fascicle lengths which is removed if fascicle strains are considered (Figure 2C). These data show that ankle joint rotations resulted in the greatest changes in fascicle length (and hence strain), during both lengthening and shortening phases of the movement cycle. The effect was particularly evident in the distal muscle region, where strains were higher and changed at a greater rate in ankle compared to knee joint conditions during both phases of the movement (Paired *T*-tests: Shortening *p* = 0.008; Lengthening *p* < 0.001). The greater fascicle strains in the distal region also coincided with lower pennation and significant differences in the rate of change in pennation also occurred between conditions during both movement phases (Shortening: Paired *T*-Test *p* = 0.006; Lengthening: Mann Whitney *p* = 0.041).

Although not as large an effect, similar differences were also found in the proximal muscle region, with greater fascicle strains and smaller pennation occurring during ankle joint rotation. Therefore, in both proximal and distal regions knee joint rotation resulted in very small changes in fascicle length (and strain) (Figure 2).

### MTU gearing

**Figure 3:**
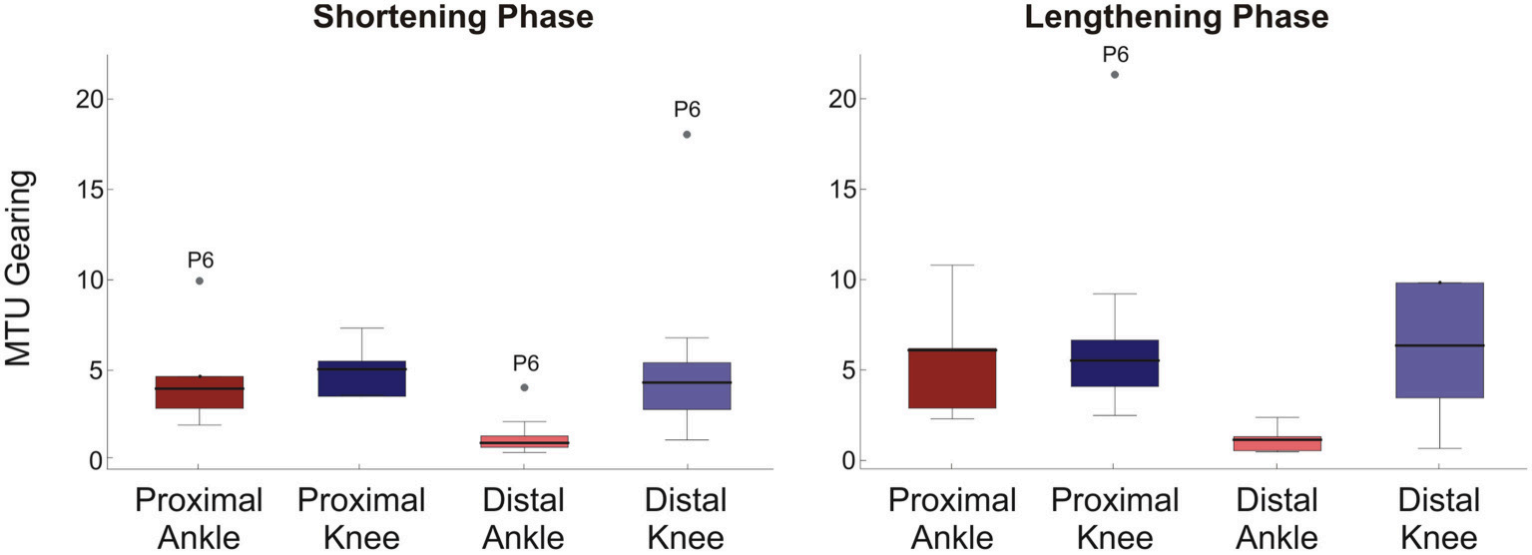
**Box plots of MTU gearing for each condition/muscle region**. Left panel shows shortening phase, right panel lengthening phase. 95% confidence intervals (whiskers, calculated excluding outliers); 25 and 75% quantiles (box edges); median (thick line). Participant number is indicated by outlier points. To provide a suitable scale, three outliers are not shown: one proximal ankle shortening phase, two distal knee lengthening phase.

MTU gearing values were typically greater than 1, with the median ranging to about 6 (Figure 3). This represents changes in fascicle length of 20–3 mm respectively and reflects that fascicle length, generally, changed less than the 20 mm MTU length change investigated. As would be predicted from the differences in Δ*L*_f_, the biggest difference in gearing values occurred in the distal muscle region with significance occurring in the statistical analysis (Mann Whitney U-Tests: shortening phase *p* = 0.003, lengthening phase *p* = 0.004). No significant differences in MTU gearing between rotations occurred in the proximal muscle region (Mann Whitney U-Tests: *p*≥0.13 all cases). In all muscle regions MTU gearing values were similar between shortening and lengthening phases of the movement cycle.

## Discussion

The purpose of this study was to test whether the extent and velocity of fascicle length change, and hence MTU gearing, within proximal and distal regions of the passive MG muscle varied depending on the joint at which the motion occurred. The results revealed passive MTU gearing values greater than 1, with striking differences in the velocity of fascicle length change (Figure 2A) and MTU gearing (Figure 3) between ankle and knee joint conditions for the same change in MTU length.

The MTU gearing values greater than 1 (Figure 3) indicate that the muscle fascicles in the MG changed length to a lesser extent than the 20 mm change in MTU length studied within the experimental protocol. Active MG MTU gearing during cycling has been reported to range between 1 and 2 (Wakeling et al., 2011). The larger gearing values reported here indicate that the muscle fascicles underwent relatively small length changes during the passive uni-articular rotations studied. This is in broad agreement with Herbert et al. (2002) who showed that changes in MG fascicle length reflected approximately 25% of the total MTU length change, indicating a passive MTU gearing value of 4. Here, we have additionally shown that passive MG MTU gearing values can vary and are influenced by the joint at which MTU length change is imposed.

The gearing values greater than 1 indicates that fascicles operated at lower velocities, and hence underwent smaller length changes, than the MTU during the passive joint rotations (Figures 2A, 3). Two possible explanations for this phenomenon are: (i) rotation of the muscle fascicles and (ii) introduction of slack into the tendons. Based on trigonometry, fascicles would be required to rotate to pennation angles beyond 50° to accommodate the small changes in fascicle length recorded. Maximal voluntary contractions can lead to high pennation (Maganaris et al., 1998b), however such values did not occur during the passive movements studied here, where changes in pennation were strikingly small (Figure 2B). Fascicle rotation does not, therefore, fully explain the differences between fascicle and MTU length changes observed.

When considering the potential contribution of slack in tendinous structures, it is important to consider that MTU gearing (*V*_MTU_/*V*_f_) is the product of two factors: (i) the ratio of muscle belly to muscle fascicle velocity (*V*_b_/*V*_f_) and (ii) the ratio between MTU and muscle belly velocity (*V*_MTU_/*V*_b_) (Wakeling et al., 2011). This means that displacements of the muscle belly and the MTU can be uncoupled as a result of the contribution of the tendon. Although difficult to predict without measures of force, it could be expected that, due to its location and its relatively larger moment arm (Table 1), greater slack would be introduced into the Achilles tendon during rotation of the ankle joint. If this were true, higher MTU gearing values for this condition would be predicted. This however is not seen, with lower MTU gearing found (Figure 3) resulting from the fact that the largest changes in fascicle lengths occurred during ankle joint rotation (Figure 2).

It is possible that anatomical differences of tendinous structures at the MG origin and insertion sites may influence how much of the changes in MTU length are “seen” by the muscle fascicles within each joint rotation condition. It has been reported that the long, compliant Achilles tendon contributes more than muscle fascicles to changes in MTU length imposed by ankle rotation (Herbert et al., 2002, 2011, 2014; Abellaneda et al., 2009), however little is currently reported about the potential contribution of the shorter and thicker proximal tendon at its origin (Dalmau-Pastor et al., 2014), making it hard to determine its influence. It is apparent however that neither fascicle rotation nor tendon slack can fully account for the differences in fascicle and MTU length changes seen. This suggests that additional factors, which may also explain the variation in passive MTU gearing values, should be considered. One potential factor is the influence of surrounding structures such as muscles, connective tissue, and bone.

Assuming constant muscle volume (Van Leeuwen and Spoor, 1992), it is apparent that changes in MTU length must be accompanied by changes in the cross-sectional area of the muscle belly if tendon length does not change. This means that increases and decreases in muscle cross-sectional area must correspond with shortening and lengthening of the MTU, respectively. Changes in muscle fascicle characteristics (e.g., length and pennation) will be coupled to changes in muscle belly shape that, in turn, will depend on external forces applied from surrounding structures (i.e., connective tissues, musculature, bone). The greater influence of ankle joint rotation on fascicle length and pennation could therefore reflect significant interactions with surrounding musculature and connective tissues. For example, MTU length changes imposed by ankle rotation are translated to the MG via the Achilles tendon, which is shared with the adjacent soleus muscle. The mono-articular soleus only spans the ankle joint, and should therefore not have been subject to length changes during the knee rotation condition (although see text below regarding epimuscular myofascial force transmission). Rotation of the ankle joint therefore results in shape changes in the soleus that influence the shape of the MG, an influence that does not occur (or differs) during knee joint rotation and may also be influenced by interactions that occur with the lateral head of the gastrocnemius. The potential for such interaction is supported by the fact that the largest differences in MTU gearing between rotation conditions occurred in the distal MG (Figure 3) where the greatest overlap between MG and soleus occurs (see Figure 13 in Dalmau-Pastor et al., 2014).

Interaction between adjacent muscles during passive loading at physiologically relevant muscle lengths has, to our knowledge, not been widely investigated. Passive knee joint rotation, with a fixed ankle angle, has been shown to result in displacement of soleus muscle (Bojsen-Møller et al., 2010). While comparison of two different knee/hip joint angles, achieved with a fixed ankle angle, has been shown to result in deformation of soleus muscle tissue (Yaman et al., 2013). These phenomena have been attributed to epimuscular myofascial force transmission, related to connections including collagen enforced neurovascular tracts, intermuscular septa, and compartment fascia (Bojsen-Møller et al., 2010; Yaman et al., 2013). These factors, alongside other features such as connective tissues or friction between muscle bellies, may have influenced current results, and suggests that both the active and passive dynamic interaction between adjacent muscles in physiologically intact systems needs further consideration for accurate models of muscle-tendon mechanics.

The results presented here should be considered within the context of at least one potential limitation: no measure of muscle activation was included. Given the angular velocity used within the protocol (60° s^−1^) reflex activation of MG may have occurred and influenced muscle stiffness in the two conditions. A small pilot trial revealed that for both ankle and knee joint conditions such tonic activation was low (< 5% MVC). Previous reports have suggested that for any resistive torques to be considered passive muscle activation should be below 1% MVC (McNair et al., 2002; Gajdosik et al., 2004). The effect of such low levels of activation on fascicle length changes is however unknown. As the clarity of the ultrasound images was a priority for the work presented and the space required to appropriately place and secure the two ultrasound probes to the leg limited the ability to appropriately position and securely place surface electromyography (EMG) electrodes, they were omitted from the main data collection. Fascicle characteristics were derived from fitting Fourier series to data points collected across all cycles (Figure 1B), if activity did occur in some of the trials this approach ensures that it would be likely to have minimum influence on the results, however it must be stated that, whilst we are certain the muscles were relaxed and not actively contracting, we cannot be sure that the muscles were investigated under totally passive conditions.

One unexpected feature of the results was the differences in fascicle lengths and pennation that are apparent between shortening and lengthening phases in both the proximal and distal muscle regions (Figure 2). Fascicle lengths were longer when the MTU was being “released” (i.e., shortening phase) and shorter when being stretched (i.e., lengthening phase), suggesting a form of history dependence within these passive movements (with analysis based on series of cycles recorded across multiple trials). Hysteresis in passive length-tension curves has previously been reported in isolated canine internal abdominal muscles (Hwang et al., 2005) and rabbit masseter muscle (Van Eijden et al., 2002). Hysteresis is also reported to occur in human Achilles tendon, although discrepancies between *in vitro* and *in vivo* measures have led to questions regarding the potential of ultrasound imaging and tracking methodologies to introduce variability and bias into these measures (Finni et al., 2013). Whether the differences between shortening and lengthening phase values for fascicle length and pennation angle found here reflect physiological properties or aspects of the ultrasound imaging approach is therefore unclear and would require further investigation.

Dissociation between changes in MTU and fascicle length are well documented during active contractions (Wakeling et al., 2011; Randhawa et al., 2013) as well as during locomotion (Fukunaga et al., 2001) and jumping performance (Kawakami et al., 2002) in humans, with proposed benefits for mechanical power output and economic force production. Here we show that a similar dissociation can occur during passive joint movements. This phenomenon may play an important functional role by altering the region of the force-length relationship over which a fascicle operates when first activated during a locomotor task. In addition we show that, within a bi-articular muscle, changes in fascicle length are affected by the joint at which the motion occurs. These findings suggest that mechanical constraints may be imposed on a muscle by the kinematics of the motor task, with the ability of a muscle to generate force influenced by the site at which MTU length changes are imposed. This undermines current assumptions of a constant relationship between muscle force/function and MTU length, which are often made in conceptual models of muscle-tendon mechanics. These results indicate the potential for more anatomically realistic muscle models to be implemented within musculoskeletal simulations of movement. This is increasingly important for investigating the mechanical and neuromuscular consequences of abnormal gait patterns/changes in muscle properties (e.g., spasticity) as well as to inform development of effective treatment and rehabilitation protocols (e.g., including robotics) in those with compromised motor abilities.

## Author contributions

EH, JW, and TD contributed to the conception and design of the work; EH and TD acquired and analyzed these data; EH, JW, and TD interpreted data for the manuscript. EH, JW, and TD drafted the work and/or provided critical revisions for important intellectual content and provided final approval of the version to be published. EH, JW, and TD agree to be accountable for all aspects of the work in ensuring that questions related to the accuracy or integrity of any part of the work are appropriately investigated and resolved.

### Conflict of interest statement

The authors declare that the research was conducted in the absence of any commercial or financial relationships that could be construed as a potential conflict of interest.
